# Endogenous glutamine decrease is associated with pancreatic cancer progression

**DOI:** 10.18632/oncotarget.20545

**Published:** 2017-08-24

**Authors:** Cecilia Roux, Chiara Riganti, Sammy Ferri Borgogno, Roberta Curto, Claudia Curcio, Valeria Catanzaro, Giuseppe Digilio, Sergio Padovan, Maria Paola Puccinelli, Monica Isabello, Silvio Aime, Paola Cappello, Francesco Novelli

**Affiliations:** ^1^ Center for Experimental Research and Medical Studies, Città della Salute e della Scienza di Torino, 10126 Turin, Italy; ^2^ Department of Molecular Biotechnology and Health Sciences, University of Turin, 10126 Turin, Italy; ^3^ Department of Oncology, University of Turin, 10126 Turin, Italy; ^4^ Department of Science and Technologic Innovation, Università del Piemonte Orientale “A. Avogadro”, 15121 Alessandria, Italy; ^5^ Institute for Biostructures and Bioimages (CNR) c/o Molecular Biotechnology Center, 10126 Turin, Italy; ^6^ Clinical Biochemistry Laboratory, Città della Salute e della Scienza di Torino, 10126 Turin, Italy; ^7^ Molecular Biotechnology Center, University of Turin, 10126 Turin, Italy

**Keywords:** PDAC, amino acid, circulating biomarkers, pancreatitis, diagnosis

## Abstract

Pancreatic ductal adenocarcinoma (PDAC) is becoming the second leading cause of cancer-related death in the Western world. The mortality is very high, which emphasizes the need to identify biomarkers for early detection. As glutamine metabolism alteration is a feature of PDAC, its *in vivo* evaluation may provide a useful tool for biomarker identification. Our aim was to identify a handy method to evaluate blood glutamine consumption in mouse models of PDAC. We quantified the *in vitro* glutamine uptake by Mass Spectrometry (MS) in tumor cell supernatants and showed that it was higher in PDAC compared to non-PDAC tumor and pancreatic control human cells. The increased glutamine uptake was paralleled by higher activity of most glutamine pathway-related enzymes supporting nucleotide and ATP production. Free glutamine blood levels were evaluated in orthotopic and spontaneous mouse models of PDAC and other pancreatic-related disorders by High-Performance Liquid Chromatography (HPLC) and/or MS. Notably we observed a reduction of blood glutamine as much as the tumor progressed from pancreatic intraepithelial lesions to invasive PDAC, but was not related to chronic pancreatitis-associated inflammation or diabetes. In parallel the increased levels of branched-chain amino acids (BCAA) were observed. By contrast blood glutamine levels were stable in non-tumor bearing mice. These findings demonstrated that glutamine uptake is measurable both *in vitro* and *in vivo*. The higher *in vitro* avidity of PDAC cells corresponded to a lower blood glutamine level as soon as the tumor mass grew. The reduction in circulating glutamine represents a novel tool exploitable to implement other diagnostic or prognostic PDAC biomarkers.

## INTRODUCTION

Pancreatic Ductal Adenocarcinoma (PDAC) is the fourth leading cause of cancer mortality in Western countries, with a very low improvement of the survival rate over the last four decades [1]. To date, the only treatment with curative intent is surgery, but as a silent killer, PDAC symptoms are so well-hidden that around 85% of people are diagnosed at an advanced stage of the disease leading to poor prognosis and incidence equaling mortality [2]. For this reason, identification of new circulating diagnostic markers as well as targets for the design of novel therapies for PDAC is crucially important [1, 3, 4]. The serum marker - Carbohydrate Antigen 19.9 (CA19-9) - is widely accepted as a prognostic PDAC marker, but due to its poor sensitivity and specificity is unsuitable for early detection of PDAC [4, 5]. In the last 20 years, many biomarkers have been studied for potential use in PDAC diagnosis, but none of them has shown clinical benefits [5].

Oncogenes and tumor suppressors as K-Ras and TP53 are highly integrated in metabolic processes [6, 7]. In some tumors, besides glucose metabolism, glutamine is a key substrate required for anabolic growth of mammalian cells [8, 9]. In K-Ras mutated tumor cells, glutamine is one of the major source of carbon for ATP production, nucleotide and protein biosynthesis and regulator of redox balance [10, 11]. Due to the importance of glutamine for cell survival and proliferation, many newly-developed diagnostic imaging techniques have focused on glutamine instead of glucose [12, 13, 14].

In this study, we aimed at the identification of a handy tool for the evaluation of circulating glutamine. Firstly, we assessed glutamine consumption *in vitro* in PDAC and non-PDAC tumor cells and control human pancreatic cells. The former showed a higher glutamine uptake that we correlated with an increased activity and expression of glutamine pathway-related enzymes. Finally, we measured the levels of circulating free glutamine, by HPLC [15, 16] or MS [17], in both an orthotopic and a genetically engineered mouse (GEM) model of PDAC [18]. In both models, a decrease of glutamine blood levels was detected and this was associated with the growing tumor mass and the progression from pancreatic intraepithelial lesions (PanIN) to invasive PDAC. These findings indicating that the decrease of circulating glutamine is a feature specifically associated to PDAC progression, may efficiently implement other PDAC diagnostic biomarkers.

## RESULTS

### PDAC cells display an increased *in vitro* glutamine uptake

We have measured the uptake of glutamine in murine [18] and human PDAC and non-PDAC cell lines, derived from human breast adenocarcinoma and a human lung adenocarcinoma, and a pancreatic ductal epithelial cell line (HPDE) used as control. Such tumor cells can have either a single K-Ras or TP53 mutation, or combined K-Ras and TP53 mutations (Supplementary Table 1).

**Figure 1:**
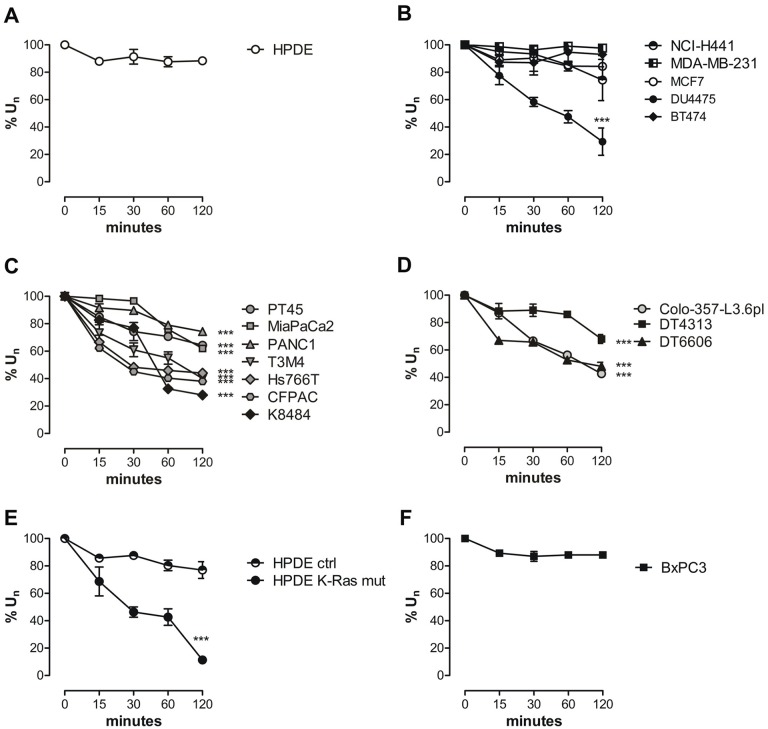
PDAC cells exhibit an increased *in vitro* glutamine uptake **(A-F)** Glutamine uptake was evaluated as the amount of 2-^15^N-Gln lasting in culture media from 0 to 120 min. The uptake is expressed as % of glutamine in culture medium. Results are represented as mean±SEM of triplicates of two independent experiments. SEM less than 10% of the value is not visible in the graph. Curves were compared by two-way ANOVA, ^***^ P <.001.

Cells were incubated with medium supplemented with glutamine and its uptake was kinetically followed at several time points on the basis of residual glutamine in the medium. The normalized percentage uptake %U_n_ is plotted against incubation time in Figure 1. HPDE cells showed a very low uptake of glutamine at 15 min that lasted until 120 min (Supplementary Table 1 and Figure 1A), with a very little delta (Δ^120-0^ %U_n_) of uptake between 0 and 120 min (Supplementary Table 1). Non-PDAC cancer cells NCI-H441 and MDA-MB-231, both featuring the combined K-Ras and TP53 mutations, also showed a low rate of uptake, being similar to that of HPDE cells (Supplementary Table 1 and Figure 1B). The same trend was observed in other breast cancer cells, with exception of DU4475 cells, derived from a Triple Negative Breast Cancer (TNBC) (Supplementary Table 1 and Figure 1B). By contrast, in all human and murine PDAC cells mutated in K-Ras only or in the combination with TP53 there was a significant and lasting glutamine uptake from 0 min to 120 min, (Supplementary Table 1 and Figure 1C-1D). These findings are in agreement with our and others studies demonstrating that oncogenic K-Ras plays a critical role in affecting glutamine metabolism specifically in PDAC [11, 19]. To confirm K-Ras role in glutamine uptake we assessed the glutamine uptake in HPDE cells expressing the mutated K-Ras oncogene. A significantly higher %U_n_ compared to HPDE cells expressing the control vector was observed (Figure 1E). The minimal role of TP53 in affecting glutamine uptake was demonstrated by the evidence that BxPC3 cells showed a similar uptake rate of unmutated HPDE cells (Figure 1F).

### PDAC cells rely on glutamine to support nucleotide biosynthesis and ATP production

**Figure 2:**
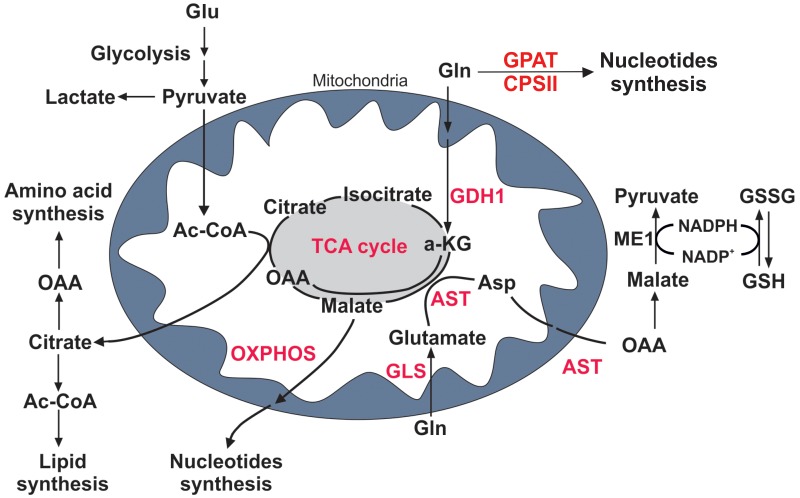
Glutamine metabolism in PDAC cells Cartoon representing metabolic pathways in proliferating cells. Glucose (Glu) and glutamine (Gln) are metabolized into critical intermediates that are necessary for energy balance and cellular growth, such as nucleotides, amino acids and lipids. Key enzymes in glutaminolysis are discussed in the text (shown in red). A-KG, α-ketoglutarate; OAA, oxaloacetate; Ac-CoA, acetyl coenzymeA; Asp, aspartate; CPSII, carbamoyl phosphate synthetase II; GSH, glutathione; GSSG, oxidized glutathione; GLS, glutaminase; GPAT, glutamine amido phosphoribosyl transferase; ME1, malic enzyme 1; AST, glutamic-oxaloacetic transaminase; GDH, glutamate dehydrogenase; TCA cycle, tricarboxylic acid cycle.

**Figure 3:**
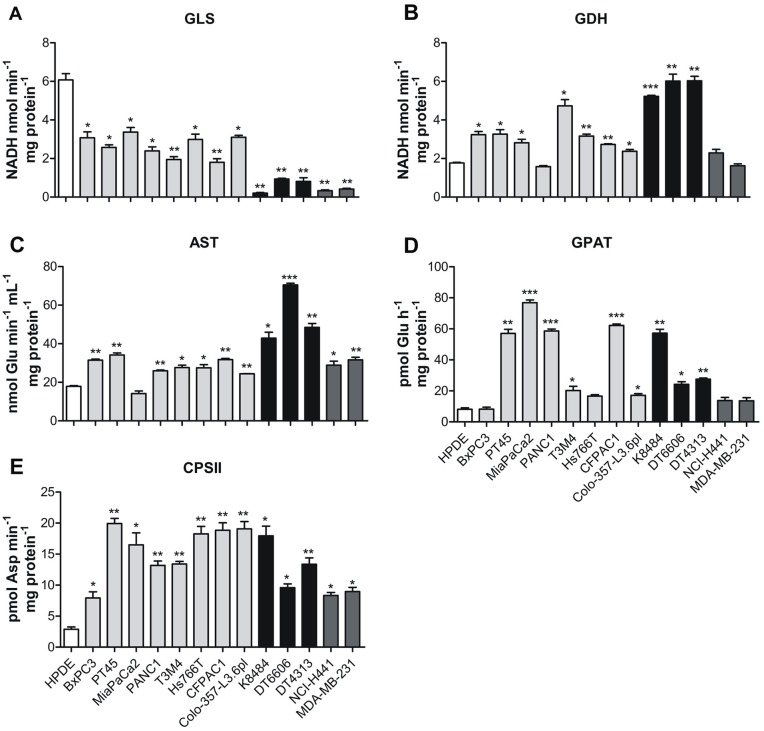
PDAC cells rely on glutamine to support nucleotide biosynthesis **(A-E)** Analysis of the enzymatic activity of: GLS (A), GDH (B), AST (C), GPAT (D) and CPSII (E) in control human pancreatic cells HPDE (white bar), human pancreatic tumor cells PT45, MiaPaCa2, PANC1, T3M4, Hs766T, CFPAC1, Colo-357-L3.6pl (light grey bars), murine pancreatic tumor cells K8484, DT6606, DT4313 (black bars) and non-PDAC cells NCI-H441 and MDA-MB-231 (dark grey bars). Activity is expressed as nmol of NADH consumed in 1 min per mg of protein for GLS and GDH; nmol of glutamate consumed in 1 min in 1 ml per mg of protein for AST; pmol of glutamate consumed in 1 hour per mg of protein for GPAT and pmol of carbamoyl aspartate in 1 min per mg of protein for CPSII. Results are represented as mean±SEM of triplicates of two independent experiments. ^*^ P <.05; ^**^ P <.01; ^***^ P <.001 values different from those obtained with the HPDE cell line.

The increased glutamine uptake observed in PDAC cells led us to analyze the activity of the enzymes involved in glutaminolysis, assuming that PDAC cells subsequently catabolize all the internalized glutamine. Glutamine catabolism begins with glutaminase (GLS), an amidohydrolase that catalyzes the conversion of glutamine to glutamate (Figure 2) [20, 21]. We observed that the overall enzymatic activity of GLS was significantly reduced in all PDAC and non-PDAC cell lines tested, compared to HPDE cells (Figure 3A). These results suggest that GLS is not the key glutamate-producing enzyme in PDAC cells.

Downstream of glutaminase, glutamine catabolism can proceed by means of the mitochondrial matrix enzyme glutamate dehydrogenase (GDH), which generates α-ketoglutarate, or via aspartate transaminase (AST/GOT), which exists in cytoplasmic and mitochondrial forms, GOT1 and GOT2, respectively (Figure 2) [4, 11]. AST catalyzes the transfer of the amino group from glutamate to α-ketoglutarate, to form aspartate from TCA cycle-derived oxaloacetate. Both these metabolic pathways were shown to be increased in almost all of the studied PDAC cell lines compared to control human and non-PDAC cell lines (Figure 3B–3C). The enzyme mRNA expression was evaluated by qRT-PCR analysis, confirming the decrease of GLS expression in tumor cell lines compared to HPDE (Supplementary Figure 1A), and the increase of mRNA levels of both GDH1 and GDH2 in PDAC cell lines compared to non-PDAC and control human cells (Supplementary Figure 1B-1C).

The gain of glutamate-catabolic enzymes and the simultaneous reduction of GLS activity led us to investigate other metabolic pathways involved in the production of glutamate. As glutamine serves as an essential nitrogen donor in purine and pyrimidine synthesis [7], the activity of glutamine amidophosphoribosyl transferase (GPAT) and carbamoyl phosphate synthetase II (CPSII), involved in the first steps of purine and pyrimidine biosynthesis respectively, were higher in all PDAC cell lines compared to non-PDAC and control human cells (Figure 3D–3E). At the same extent also AST, GPAT and CPSII mRNA expression levels were higher in PDAC cell lines compared to non-PDAC and control human cells (Supplementary Figure 1D-1F). These results suggest that PDAC cell lines have different glutamine metabolism compared to the non-PDAC cell lines, despite the presence of mutated K-Ras.

Furthermore, the increased expression of GDH1 and CPSII was also observed by immunohistochemistry (Supplementary Figure 2), confirming a higher presence of both enzymes in the pancreas of both orthotopic and GEM models of PDAC in comparison to WT mice.

**Figure 4:**
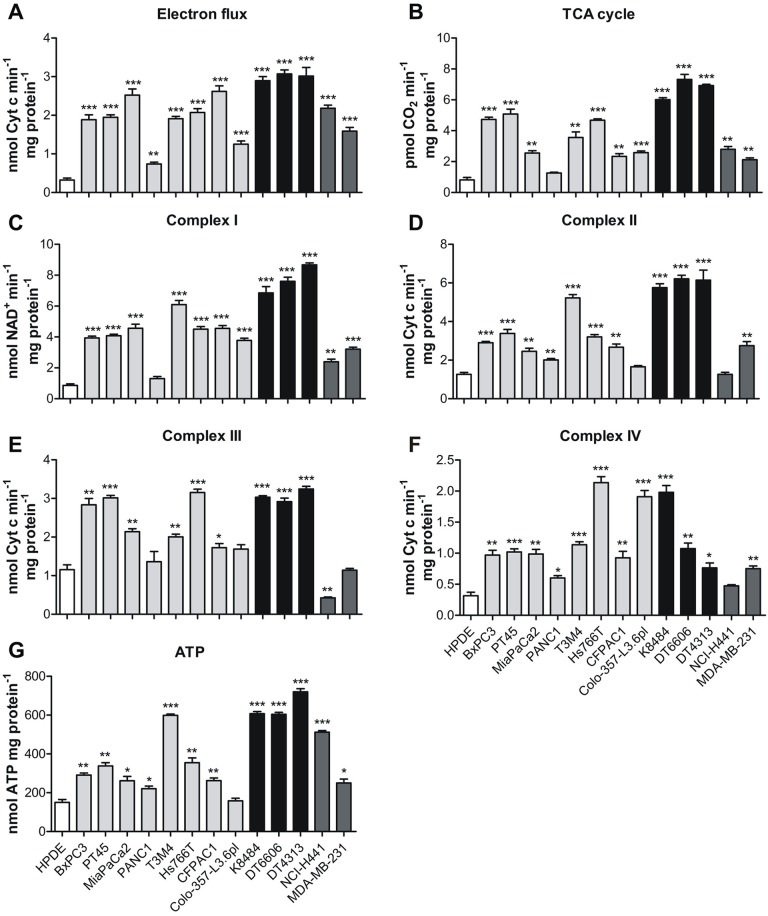
Oxidative phosphorylation is increased in PDAC cells **(A)** The TCA cycle rate was evaluated by measuring CO_2_ emission after radiolabeling cells with [1-^14^C] acetylcoenzyme A in human HPDE cells (white bars) and human PDAC cell lines (light grey bars), murine (black bars) PDAC cell lines and non-PDAC cells line (dark grey bars) expressed as pmol of CO_2_ consumed in 1 min per mg of protein. **(B-F)** Analysis of the global electron flux (B)and of the mitochondrial respiratory chain complexes I-IV activity (C-F) that is expressed as nmol of NAD^+^ produced in 1 min per mg of mitochondrial protein for complex I, nmol of reduced Cytc in 1 min per mg of mitochondrial protein for the global electron flux and for complexes II-III and nmol of oxidized Cytc in 1 min per mg of mitochondrial protein for complex IV. **(G)** Analysis of ATP production, expressed as nmol of ATP consumed per mg of protein. All graphs represent mean±SEM of triplicates. ^*^ P <.05; ^**^ P <.01; ^***^ P <.001 significance values, compared to those obtained with the HPDE cell line.

In addition, the TCA cycle rate was significantly increased in all PDAC and non-PDAC cell lines compared to HPDE control cells (Figure 4A). Consequently, the global electron flux and the activity of each mitochondrial complex were increased (Figure 4B–4F). This up-regulation that led to a strong increase in ATP synthesis supporting all the above-mentioned biosynthetic reactions is not so unexpected in all tumor cells (Figure 4G).

### PDAC cells utilize the ASCT2 antiport to regulate glutamine uptake

**Figure 5:**
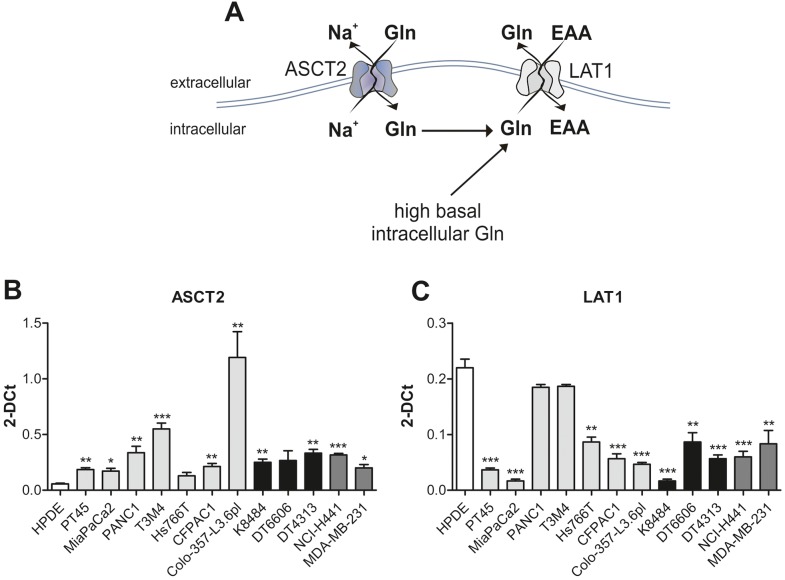
PDAC cells utilize the ASCT2 antiport to regulate glutamine uptake **(A)** Schematic representation of ASCT2 and LAT1 transmembrane proteins **(B-C)** mRNA expression analysis of ASCT2 (B) and LAT1 (C) in HPDE (white bar), human (light grey bars) and murine (black bars) PDAC cell lines and non-PDAC cells (dark grey bars). Results are represented as mean of 2-DCt±SEM of triplicates of two independent experiments ^*^ P <.05; ^**^ P <.01; ^***^ P <.001 significance values, compared to those obtained with the HPDE cell line.

As we reported an increased glutamine uptake and metabolism in PDAC cells, we wondered which transmembrane protein was responsible for its internalization. We focused on two transporters already described to be correlated to PDAC [22, 23], namely AlaSerCys Transporter 2 (ASCT2) (Figure 5A) [24] and the Light subunits of Amino acid Transporters 1 (LAT1) (Figure 5A) [25]. The former is the major glutamine importer whereas the latter is a glutamine exporter.

An overall up-regulation of ASCT2 mRNA levels and a simultaneous down-regulation of LAT1 expression occurred in most of PDAC cell lines compared to HPDE cells (Figure 5B–5C).

### Circulating glutamine decrease correlates with PDAC progression rather than inflammation or diabetes

**Figure 6:**
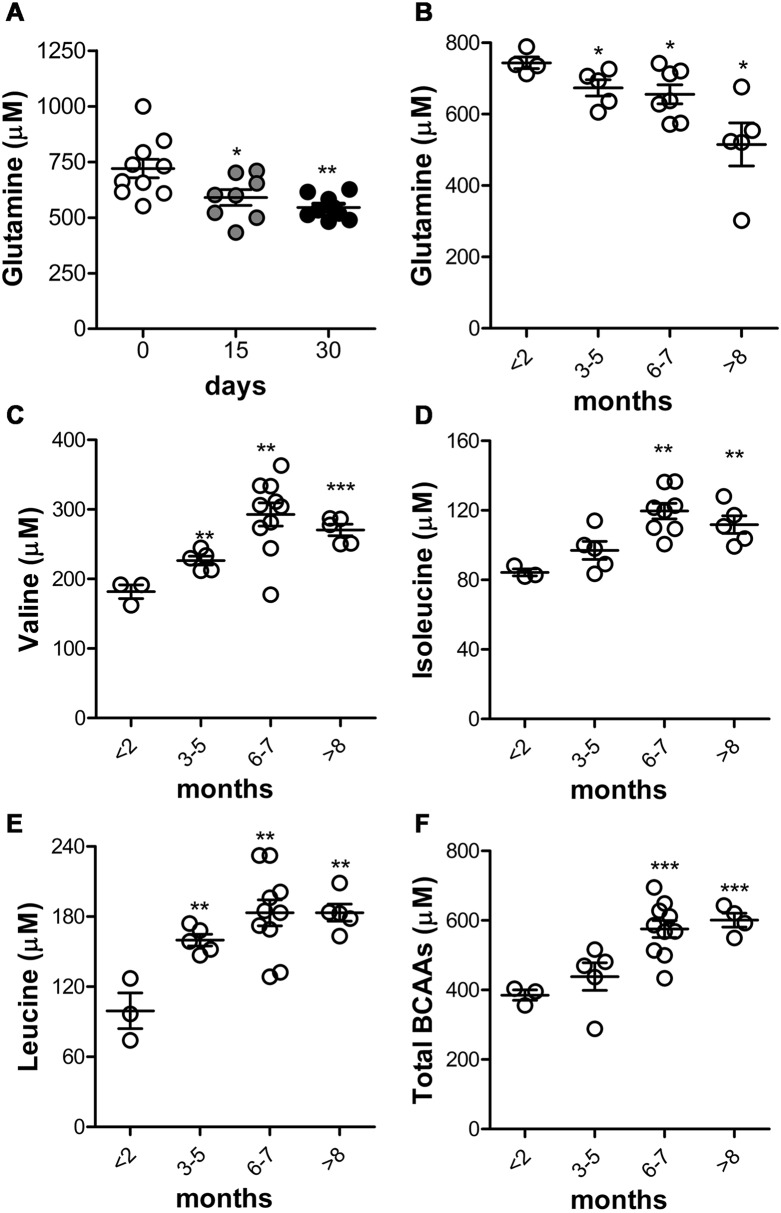
Glutamine decrease and BCAAs increase are linked to PDAC progression in KC mice **(A)** C57BL/6 mice were injected orthotopically into the pancreas with K8484 cells. Total blood glutamine concentration was evaluated using an HPLC instrument at the indicated time points. Results are represented for each mouse (10 mice/each time point) and mean±SEM are indicated. **(B-F)** Analysis of total glutamine (B), valine (C), isoleucine (D), leucine (E) and total branched-chain amino acids concentration (F) in KC mice (3-10 mice/group) at the indicated time points compared to control group (mice <2 months old). ^*^ P <.05; ^**^ P <.01; ^***^ P <.001 values at days 15 and 30 significantly different from those measured at day 0, and KC and WT mice older than 2 months compared to mice less than 2 months old.

To verify that the increased glutamine uptake and metabolism in cultured PDAC cell lines was not an artifact due to the *in vitro* culture conditions [26], we stepped into *in vivo* models. We speculated that the endogenous circulating glutamine levels were decreased *in vivo* during PDAC growth as a result of its increased uptake and consumption by tumor cells. To this end, K8484 cells were orthotopically injected into the pancreas of C57BL/6 mice, and endogenous circulating glutamine levels were assessed in parallel with the tumor mass. Hundred thousand of K8484 mixed with matrigel are able to give a mass of around 1 gr of weight by 28 days when injected into the pancreas of syngeneic mice [27]. Endogenous circulating glutamine levels were assessed at 0, 15 and 30 days after injection by HPLC [15, 16], and MS [17]. When tumor grew (Supplementary Figure 3A), circulating glutamine levels significantly decreased (Figure 6A–Supplementary Figure 3B). Such decrease was consistently maintained until the end of the experiment (Figure 6A–Supplementary Figure 3B).

To further validate the correlation between PDAC onset and progression and the reduction in circulating glutamine, we measured it in both female and male GEM mice carrying single-mutated Kras^G12D^ (KC), ranging from 1.5 to 9 months of age. As shown in Figure 6B–Supplementary Figure 3C, in 3-month-old mice, a significant reduction in the blood glutamine levels was observed. This reduction was much more pronounced in 6-month-old mice and still observed in 9–month-old mice. Remarkably, the reduction was observed as early as PanIN were detectable and independent from the mouse gender. To rule out aging as a possible factor affecting free glutamine levels in blood [28, 29], we measured it in healthy WT mice and there was no glutamine decrease at all as shown in Supplementary Figure 3D. This confirms that the decrease of circulating glutamine is a feature-associated to PDAC onset and progression. As it has been noticed that an higher plasmatic level of BCAA correlated with an higher risk of PDAC development [30, 31], we quantified BCAAs in KC mice. Valine (Figure 6C), Isoleucine (Figure 6D) and Leucine (Figure 6E) were significantly increased when evaluated both in single and all together (Figure 6F).

**Figure 7:**
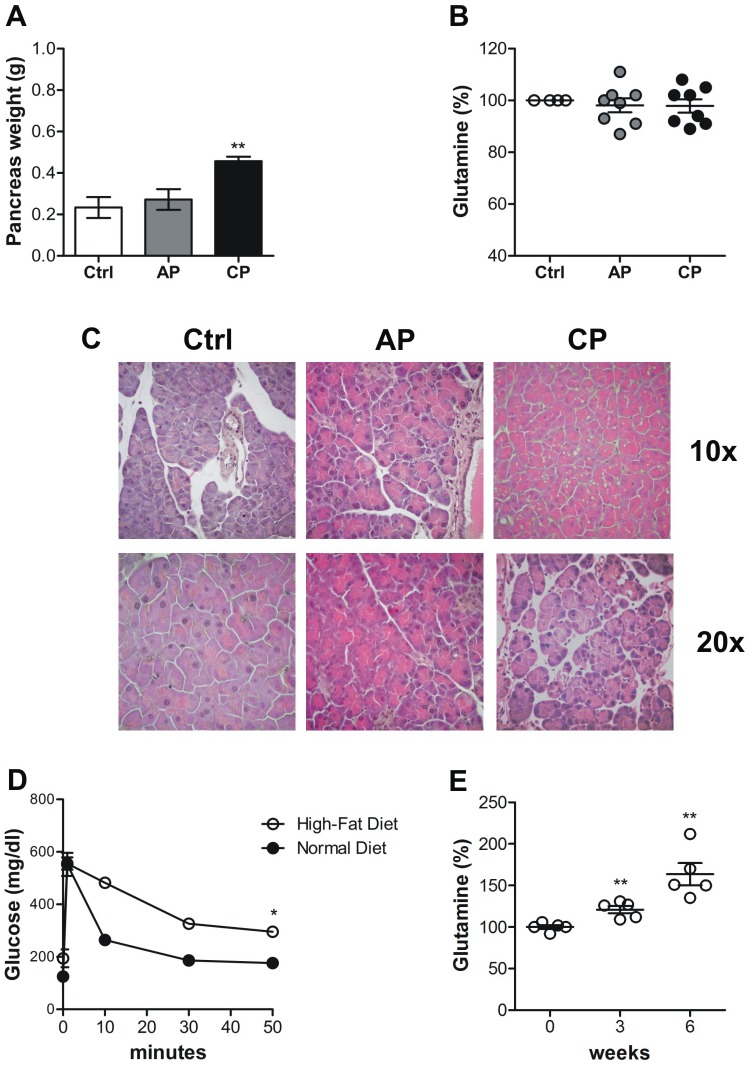
The decrease in circulating glutamine does not correlates with inflammation **(A-C)** Acute (AC) and Chronic Pancreatitis (CP) were induced in C57BL/6 mice through several injections of caerulein. A. Pancreases were collected and weighed from PBS-treated control and AC- and CP-affected mice. B. Graph represents percentage of blood glutamine compared to control group after induction of pancreatitis (5-8 mice/time point). Mean±SEM are indicated. E. H&E staining of pancreas sections of control mice, and acute and chronic pancreatitis-affected mice. **(D)** Plasma glucose concentrations during the intravenous glucose tolerance test (1 g/kg) in mice fed with Normal Diet or with High-Fat Diet for six weeks. Blood was sampled at 0, 1, 10, 30 and 50 minutes and glucose concentration is expressed as mg/dl. **(E)** Graph represents percentage of blood glutamine of mice before HFD feeding (time 0) versus 3 and 6 weeks of HFD feeding (5 mice/time point). Mean±SEM are indicated. ^*^ P <.05; ^**^ P <.01; ^***^ P <.001 values showing a significant difference of AP and CP compared to the control group for the pancreatitis model and HFD-fed mice compared to time 0.

Orthotopic procedures may cause inflammation and in humans, onset of PDAC can be associated with long-standing chronic pancreatitis [32]. To rule out the involvement of inflammation in the modulation of the blood glutamine level, we compared endogenous glutamine variation in C57BL/6 mice before and after caerulein–induced acute and chronic pancreatitis. As previously reported [33, 34], after the induction of acute pancreatitis there were no differences in pancreas weight (Figure 7A), although the histological analyses indicated tissue damage in the caerulein-treated mice but not in the PBS-treated group (Figure 7C). Repeated injections of caerulein (4-week treatment), however, induced an increase in pancreas weight (Figure 7A), and marked morphological changes with an alteration in acinar units due to increased space between neighboring cells, swollen cytoplasm and more centrally-located nuclei (Figure 7C). Although some variability among mice, no significant variations in the amount of circulating glutamine were observed (Figure 7B). Therefore, the reduction in circulating glutamine was dependent on PDAC transformation rather than its associated inflammation. Since there is a strong correlation between type 2 diabetes (T2D) and PDAC [35], to verify that circulating glutamine levels were not affected by diabetes onset, we set up a T2D mouse model [36, 37]. The model has shown to be accompanied by insulin resistance, as determined by intravenous glucose tolerance tests (IVGTT) 6 weeks after a High-Fat Diet (HFD) regimen (Figure 7D), but interestingly the circulating glutamine levels after 3 and 6 weeks of HFD resulted significantly increased (Figure 7E).

## DISCUSSION

The essential requirement of proliferating cells for glutamine was described by Eagle, who observed that glutamine consumption rate of many tumor cells exceeded the consumption of any other amino acid by 10-fold [38, 19]. Among the other energy fuels, glutamine is the most abundant amino acid in the blood and the main donor of nitrogen. PDAC cells are termed as “glutamine-addicted” as their glutamine demands are known to exceed the physiological need of this metabolite [4, 39]. In the current study, we exploited a non-invasive tool for assessing the total glutamine uptake in PDAC mouse models, exploring the hypothesis that the increased glutaminolysis was maintained *in vivo*, thus revealing reduction of circulating glutamine levels. A recent published work proposed the use of circulating metabolites, in particular BCAA, as an indication of future PDAC diagnosis [30]. Mayers et al., indeed, observed that an early event of PDAC progression related to the cachexia, is their increased plasma levels [31]. Another group proposed as PDAC diagnostic and prognostic tool the evaluation of proteins present in tumor cell-derived exosomes, one for all glypican1 [40, 41]. In our study we exploited two minimally-invasive methods to assess the levels of circulating glutamine: a semiquantitative MS [17] and a quantitative HPLC method [15, 16]. Of note, the latter is already used in clinical contexts to measure amino acid profiles for diagnosis and monitoring of metabolic diseases [15, 16].

As proof-of-concept, we evaluated the *in vitro* glutamine uptake rate of a large set of PDAC cell lines by using the 2-^15^N-Gln isotopomer. We observed a significant increase in glutamine uptake by all PDAC cells assessed. Many oncogenes or tumor suppressor genes are involved in modulating the metabolic state of cells: c-Myc [42] and mTORC1 [43] have been linked to enhanced glutamine anaplerosis for fueling growth and proliferation by activating GDH. Glutamine metabolism is also regulated by hypoxia through HIF-1 activation [44]. In mutated K-Ras PDAC, glutamine supports cell growth [11] by a non-canonical pathway: the conversion of glutamine into oxaloacetate and subsequently into malate and pyruvate rather than alpha-ketoglutarate. On the other hand, TP53 mediates tumor suppression through induction of GLS2 expression to fuel GSH synthesis in response to reactive oxygen species (ROS) [45]. We did not observe any differences in glutamine uptake by PDAC cells with mutated K-Ras alone or in combination with mutated TP53 (Supplementary Table 1). Moreover, even if only one tumor cell line showing the presence of mutated TP53 alone was analyzed, this latter seems to be no sufficient to elicit a glutamine-dependent phenotype. These results highlight that K-Ras mutations drive the increase of glutamine uptake by PDAC independently from the mutational status of TP53. This observation was also demonstrated by the higher uptake of glutamine evaluated in human pancreatic duct epithelial cells forced to express the mutated K-Ras oncogene compared to the control vector expressing cells. In our study, both breast and lung cancer cells displayed a very low *in vitro* glutamine uptake with exception of DU4475 cells, which are derived from a TNBC. Breast cancer cells are indeed heterogeneous and may be also subdivided according to their glutamine addiction independently from K-Ras mutation [46], with TNBC showing increased glutamine dependency compared to Triple Positive Breast Cancer (TPBC) [47]. The large set of PDAC cells with similar trend confirms that generally PDAC is more “glutamine addicted” than other cancers [46, 48].

The increased *in vitro* glutamine uptake by PDAC cells is to satisfy their glutamine needs. Proteomic analysis identified several enzymes involved in glutaminolysis in the PDAC cells, and revealed an increased production of glutamine-derived nucleotides through GPAT and CPSII enzymes [4, 19, 49]. This results in an increased glutamate pool that is metabolized by GDH to α-KG [50] and by AST pathway to aspartate to regulate K-Ras-dependent redox stress [11]. Glutamine catabolism can also proceed via GLS, producing glutamate and ammonia [50]. In mammals, the two major isoforms are linked to different pathways for sustaining cell proliferation and survival: GLS1 expression is enhanced by c-Myc [42], it favors cell survival and proliferation and promotes the entry of glutamine into the TCA cycle [50]. By contrast, GLS2 expression is regulated by TP53, it increases cellular antioxidant activities and regulates energy metabolism [51]. Here, all the PDAC cells analyzed showed an increase in GPAT and CPSII activities, mRNA and protein expression to produce nucleotides and glutamate, in GDH activity mRNA and protein expression to metabolize glutamate and in AST activity to produce aspartate to fuel the TCA cycle. By contrast, GLS activity and mRNA levels decreased in most of PDAC cells. Our results suggest that glutamine catabolism is shifted from GLS to GPAT and CPSII, to satisfy nucleotide demand. In non-PDAC cells, we observed a marked reduction of GLS, GDH, GPAT and CPSII activity, highlighting that the glutamine metabolism through GPAT/CPSII and GDH is typical of PDAC cells and accounts for the increased glutamine uptake.

Rapidly growing cells tend to use the carbon skeleton of glutamine as a respiratory substrate [7, 20]. As expected, in all the analyzed PDAC cells, we observed an upregulation of the mitochondrial oxidative phosphorylation, which led to an increase of the ATP pool [50] to support proliferation and survival.

Most cells can synthesize glutamine *de novo*, although tumor cells being in rapid growth take up as many nutrients as possible to satisfy increased metabolic requirements [22]. Therefore, is of advantage to use transporters to maximize nutrition incorporation. In PDAC cells, glutamine is mainly transported by ASCT2 and LAT1 [23]. Although it has been reported that high expression of ASCT2 and LAT1 may represent a marker of poor prognosis in PDAC [24, 25], in all PDAC cells assessed, only ASCT2 was up-regulated at the mRNA level while LAT1 expression was down regulated. This preferential use of ASCT2 could be attributed to the need of protein synthesis through glutamine [22]. In addition, LAT1 mRNA reduced expression could be due to a different post-transcriptional *in vitro* stabilization or to the absence in cultured conditions of tumor stroma that might lead to its expression to compensate the nutrient shortage.

To translate the *in vitro* observation into the clinical practice, we exploited an orthotopic and autochthonous mouse models of PDAC. Both C57BL/6 mice, injected with syngeneic PDAC cells, and GEM mice displayed lower levels of circulating glutamine as early as tumors appeared and progressed. GEM mice [18] showed a reduction in plasmatic glutamine levels, paralleled with the increase in BCAAs levels, from PanIN to invasive PDAC compared to their normal littermates of same age, indicating that these differences could not be ascribable to aging or gender, but solely to PDAC transformation and progression. Interestingly Mayers et al. [30] that described an increase in BCAAs levels did not report glutamine variation in their patients cohort or murine samples. This could be due to the short glutamine half-life that requires immediate and fast pre-analytical samples treatment.

To rule out any effect due to inflammation associated with PDAC progression [32], we induced acute and chronic pancreatitis [33] as well as T2D [35, 52]. Although mice with pancreatitis showed an increased pancreas weight and the presence of histological alterations, chronic pancreatitis did not affect circulating glutamine concentration. As T2D can be a consequence or a cause of PDAC progression, we fed C57BL/6 mice with 6 weeks of HFD. This models has, accordingly, been used in studies on pathophysiology of impaired glucose tolerance and T2D: it is characterized by fasting hyperglycemia, which contributes in reducing glucose-mediated insulin secretion and in the efficiency of glucose uptake by the insulin sensitive tissues [37, 36]. IVGTT was performed at 6 week after introduction of HFD, and HFD–fed mice were glucose intolerant and had impaired glucose-stimulated insulin secretion compared to normal diet-fed mice. Furthermore, blood samples were analyzed to test circulating glutamine levels and we found an increased amount of glutamine compared to the same mice before HFD feeding. It has been proved that L-glutamine triggers the secretion of glucagon-like peptide-1 (GLP-1) from intestinal cells of mice in response to elevated blood glucose levels, mirroring insulin's release from pancreas [53, 54]. Like insulin, GLP-1 lowers blood glucose level, therefore an increase of glutamine release in the blood of HFD-fed mice may be an attempt to auto-regulate glucose levels during T2D onset. Therefore, the reduction of circulating glutamine was due to the tumor growth. Data on *in vitro* glutamine uptake we obtained fit in well with those observed *in vivo*. Thus, the concern raised on discrepancy between *in vitro* and *in vivo* glutamine homeostatic regulation with lung cancer cells [26], does not apply to our study.

This study unveils a novel biomarker associated to PDAC, taking advantage of its increased glutamine uptake and catabolism, and an HPLC-based method to evaluate circulating glutamine levels with a minimally invasive blood collection followed by a fast and cheap pre-analytical sample treatment. Moreover, as the International Cancer of the Pancreas Screening (CAPS) Consortium has released consensus guidelines for pancreatic cancer screening for the individuals with specific mutational features defined “high-risk”, it will be compelling to develop a clinical trial to validate our data in a cohort of healthy, high-risk subjects, and PDAC patients to fast translate this method into the clinic.

## MATERIALS AND METHODS

### Cell culture

The cell lines used in this study were: CFPAC1, BxPC3, MCF7, BT494, DU4475 and MiaPaCa2 (all from ECACC), T3M4, PANC1, Hs766T, Colo-357-L3.6pl, PT45 and HPDE (all kindly provided by Dr. Paola Nisticò, Regina Elena National Cancer Institute, Rome, Italy), MDA-MB-231 (kindly provided by Dr. P. Michieli, University of Turin, Turin, Italy), NCI-H441 (kindly provided by Dr. R. Chiarle University of Turin, Turin, Italy), HPDE ctrl and HPDE K-Ras mut (all kindly provided by Dr. P. Allavena). The murine cell line K8484, isolated from a tumor arising in LSL-Kras^G12D/+^; LSL-Trp53^R172H/+^; Pdx-1-Cre (KPC) mouse [18], DT6606 and DT4313, isolated from a tumor arising in LSL-Kras^G12D/+^;Pdx-1-Cre (KC) mouse, were kindly provided by Dr. K. P. Olive (Columbia University, New York, NY). Cells were cultured at 37°C in Dulbecco's modified Eagle's medium (DMEM) (Lonza) supplemented with 2 mM L-glutamine (Gibco), 10% fetal bovine serum (FBS) (Lonza), and 50 mg/mL Gentamycin (Sigma-Aldrich) with humidified 5% CO_2_. All cell lines were tested routinely for mycoplasma contamination.

### Glutamine uptake *in vitro*

Cells were plated on poly-L-lysine (Sigma Aldrich) for 24 h in complete medium, then were washed twice with phosphate-buffered saline (PBS) and incubated at 37°C with 1 ml of EBSS buffer (1.80 mM CaCl_2;_ 5.30 mM KCl; 0.80 mM MgSO_4_; 117 mM NaCl; 26 mM NaHCO_3_; 5.60 mM Glucose; pH 7.4) with or without 1 mM 2-^15^N-L-glutamine (2-^15^N-Gln, Cambridge Isotope Laboratories). Supernatants were collected at fixed time points (0, 15, 30, 60 and 120 min), frozen and cells were detached. Protein content was measured using the CB-X protein assay kit (Cabbru). Aliquots (450 μl) of supernatants were mixed with 50 μl of EBSS buffer containing 10 mM N-Acetyl-L-Glutamine (Sigma Aldrich) as internal standard immediately prior to analysis and subjected to the analytical determination of the 2-^15^N-L-glutamine amount by MS [17]. We obtained a curve representing the uptake of 2-^15^N-L-glutamine expressed as percentage of normalized uptake *%U_n_ = 100 (1- C_t_/ C_0_) / d*, in which *C_0_* is the concentration of 2-^15^N-Gln in the medium at time 0, *C_t_* is the concentration of 2-^15^N-Gln in the medium at the considered time point and *d* is the total protein content (mg).

### Mass spectrometry analysis

A Waters instrument with a 515 HPLC pump system and a 3100 mass detector was used. The system was coupled with a quadrupole mass spectrometer equipped with an ESI ion source. The solvent was composed of acetonitrile + 0.1% HCOOH/water + 0.1% HCOOH. The flow rate was 1 ml/min and the electrospray ionization (ESI) was operated in the positive mode. Other MS parameters were as follows: capillary voltage 3kV, cone voltage 15V, source temperature 110°C, desolvation gas temperature 220°C, desolvation gas flow 500 l/h, cone flow 50 l/h. Analysis of the results was performed using the MassLynx software (Waters) (the same used for the system and data control).

### Glutamine catabolism and purine synthesis and pyrimidine synthesis

Glutamine catabolism and purine synthesis and pyrimidine synthesis were measured as reported in Capello et al. [49].

### Aspartate aminotransferase (AST) activity

Cells were washed with fresh medium, detached with trypsin/EDTA (0.05/0.02% v/v) and then washed twice with ice-cold PBS. The AST activity was measured using the Aspartate Aminotransferase Activity Assay (Sigma Aldrich), using a Synergy HT Multi-Mode Microplate Reader (Bio-Tek Instruments, Winooski, VT). The protein content of each lysate was quantified using the CB-X protein assay kit (Cabbru), and the AST activity was expressed as nmol of Glu/min/mL/mg cell proteins.

### Quantitative RT-PCR

Total RNA was extracted using the TRI Reagent (Sigma Aldrich) and reverse transcription was performed with 1 μg of total RNA using iScript cDNA synthesis kit according to the manufacturer's instructions (Bio-Rad). Quantitative RT-PCR was performed with SYBR Green dye (Life Technologies) on a Thermal iCycler (Bio-Rad). PCR reactions were performed in triplicate and the relative amount of cDNA was calculated with the comparative CT method using β-actin RNA sequences as a control for human cell lines, and glyceraldehyde 3-phosphate dehydrogenase RNA sequences as a control for murine cell lines. We did not perform the reaction on murine GDH2 because, in this organism, it is still a pseudogene.

### Tricarboxylic acid cycle (TCA) and ATP

TCA cycle and ATP were measured as reported in Capello et al. [49].

### Mitochondrial respiratory chain

Mithochondrial extracts were prepared as reported for ATP quantification [49]. A 50 μL was sonicated and used for measuring the protein content. The rate of cytochrome c (cyt c) reduction was taken as an index of the activity of mitochondria respiration, according to [55] with minor modifications. Non-sonicated mitochondrial samples (50 μg) were re-suspended in 0.59 mL buffer A (5 mmol/L KH_2_PO_4_, 5 mmol/L MgCl_2_, 5% w/v bovine serum albumin), and transferred to a quartz spectrophotometer cuvette. A volume of 0.38 mL of buffer B (25% w/v saponin, 50 mmol/L KH_2_PO_4_, 5 mmol/L MgCl_2_, 5% w/v bovine serum albumin, 0.12 mmol/L cytochrome c-oxidized form, 0.2 mmol/L NaN3) was then added for 5 min at room temperature. The reaction was started with 0.15 mmol/L NADH and was monitored for 5 min, reading the absorbance at 550 nm using a Lambda 3 spectrophotometer (PerkinElmer, Waltham, MA). Results were expressed as nmol of cyt c reduced/min/ mg mitochondrial protein. The activities of Complexes I, II, III and IV were measured spectrophotometrically as reported by [55].

### Mice

For orthotopic experiments, C57BL/6 mice (not carrying Kras and TP53 mutations and bred at the animal facility of the Molecular Biotechnology Center, University of Turin, Italy) were injected in the pancreas with 1×10^5^ K8484 cells in Matrigel (1:5; BD) and euthanized after 15 or 30 days. Blood was collected by cardiac puncture using a 22-gauge needle and a 1 ml syringe from mice previously anesthetized. Acute pancreatitis was induced in C57BL/6 mice by six hourly intraperitoneal (i.p.) injections of 50 μg/kg of caerulein (kindly provided by Dr F. Bussolino, IRCCS Candiolo, Turin, Italy). Mice were sacrificed 7 hours after the first caerulein injection. Chronic pancreatitis was induced by six hourly i.p. injections of 50 μg/kg caerulein once a week for 4 weeks. Mice were sacrificed 3 days after the final caerulein injection. GEM mice carrying single-mutated Kras^G12D^ (KC) were bred and maintained under saprophytic and pathogen-free conditions at the animal facilities of the Molecular Biotechnology Center and treated in accordance with EU and institutional guidelines. Diabetes was induced by housing C57BL/6 mice with High-Fat diet containing (w/w) 60% fat, 18% protein and 22% carbohydrate (Thermo Fisher D12492). In the sixth week mice were subjected to intravenous glucose tolerance tests as described below.

### Intravenous glucose tolerance tests

The intravenous glucose tolerance tests were performed (n=5 per test group for each test) following six weeks of High-Fat diet. Animals were studied after an overnight fast and anesthetized, a bolus of glucose (1 g/kg) was injected i.v. and blood was sampled at 0, 1, 10, 30 and 50 min for plasma glucose analyses with an Accu-CHEK Aviva device.

### Immunohistochemical staining

Pancreatic tissues from WT and KC mice at 3 and 9 months of age mice and tumors arose after orthotopic injection of K8484 cells were formalin-fixed and paraffin-embedded. Then tissues were cut into 3 μm thick slices and peroxidase activity was inhibited by immersing the slides in 3% hydrogen peroxide aqueous solution for 10 minutes. Pancreatic tissue samples were pretreated by microwave antigen retrieval using citrate buffer (Dako, Milano, Italy) and incubated with CPSII antibody (1:300, 30 minutes at room temperature, order number ab99312, Abcam, Cambridge, UK) or with pH9 buffer and stained with GDH1 (1:400, 30 minutes at room temperature, order number ab166618, Abcam). Than rabbit En Vision system (Dako) was used before diaminobenzidine tetrahydrochloride (Dako) incubation. Negative and positive controls were performed to set up the staining protocol. All slides were stained for the same antigen together with the same antigen retrieval buffer and antibody dilution. Tissues were examined in a double-blind fashion and digital images of representative area were taken.

### Preparation of murine blood samples for MS analysis

Aliquots (200 μl) of murine whole blood were collected in tubes containing heparin. Samples were deproteinized with an equal amount (200 μl) of 2 mM N-Acetyl-L-Glutamine (Sigma Aldrich) dissolved in 1:9 H_2_O-acetonitrile, vortexed, and kept on ice for 10 min. After centrifugation at 4000 rpm for 10 min at 4°C, supernatants were transferred into a clean tube. Samples were then stored at -20°C and glutamine analysis executed within two days.

### Preparation of murine blood samples for HPLC analysis

Aliquots (100-200 μl) of whole blood from mice orthotopically injected with K8484 cells, as previously described, were collected in tubes containing K_2_-EDTA. After centrifugation at 3500 rpm for 5 min at 4° C, plasma was transferred into a clean tube and deproteinized with a 5-sulfosalicylic solution (15%, w/v), vortexed for 30 sec and left at room temperature for 15 min. Samples were then stored at -80°C. Samples are diluted in 0.2M lithium citrate (pH 2.20) containing the internal standard N-valine. The amino acid analysis method was based on ion exchange chromatography with post column derivatization with Ninhydrin (Amino Acid Analyzer 30 Biochrom Ldt, UK) [15, 16].

### Tissue sample and histopathology

Mice were euthanized at the indicated time points, necropsied and examined for the presence of tumor masses. Tumor masses were fixed in 4% (v/v) neutral-buffered formalin (Sigma-Aldrich) overnight, transferred to 70% ethanol, and paraffin-embedded. For histological analysis, formalin-fixed paraffin-embedded tissue sections of 5μm were cut and stained with hematoxylin-eosin.

### Statistical analysis

Statistical analyses were performed using the unpaired Student's *t* test and two-way ANOVA with Sidak's post hoc test, as appropriate (GraphPad Prism4 software). For all experiments with error bars, SEM was calculated to indicate the variation within each experiment and data, and values represent mean ± SEM.

## SUPPLEMENTARY MATERIALS FIGURES AND TABLES



## Abbreviations

PDAC: pancreatic ductal adenocarcinoma
MS: mass spectrometry
HPLC: high performance liquid chromatography
GEM: genetically engineered mouse
KC: Kras^G12D^; Cre mice
KPC: Kras ^G12D^/Trp53^R172H^; Cre mice
CA19-9: Carbohydrate Antigen 19.9
DMEM: Dulbecco's modified Eagle's medium
FBS: fetal bovine serum
PBS: phosphate-buffered saline
EBSS: Earle's balanced salt solution
UV: ultraviolet
ESI: electrospray ionization
AST: Aspartate Aminotransferase
GPAT: glutamine amido phosphoribosyl transferase
CPSII: carbamoyl phosphate synthetase II
TCA cycle: tricarboxylic acid cycle
Glu: glucose
α-KG: α-ketoglutarate
OAA: oxaloacetate
Ac-CoA: acetyl coenzymeA
Asp: aspartate
GSH: glutathione
GSSG: oxidized glutathione
GLS: glutaminase
ME1: malic enzyme 1
GDH: glutamate dehydrogenase
BCAA: branched chain amino acids
TNBC: Triple Negative Breast Cancer
TPBC: Triple Positive Breast Cancer
HFD: High-Fat Diet
IVGTT: intra venous glucose tolerance test
T2D: Type 2 Diabetes
