# Supplementary material for: Endogenous glutamine decrease is associated with pancreatic cancer progression
Source: Oncotarget. 2017 Aug 24;8(56):95361–76. doi: 10.18632/oncotarget.20545 (PMC5707027; doi:10.18632/oncotarget.20545)
Supplement: Supplementary file 1 [file oncotarget-08-95361-s001.pdf]

Endogenous glutamine decrease is associated with pancreatic cancer progression

SUPPLEMENTARY MATERIALS

Oligonucleotide primer sequences for SybrGreen qRT-PCR

Gene	Sense	Antisense
Human ASCT2	CCGCTTCTTCAACTCCTTCA	GTAAACCCACATCCTCCATCTC
Mouse ASCT2	GGTTCTGCCTCTCATCTACTTC	CCACACCATTCTTCTCCTCTAC
Human LAT1	GTGGCCTCTTGGCTATTTCT	GTGTCTGCCTTTCTTGTCTCT
Mouse LAT1	GCTGTCGTTCAGTAGCATAGAG	GGTGATGTGCAAGTCTCAGTAG
Human GLS	AGGTGGTGATCAAAGGGTAAAG	TCCATGTCCATAGCTGACAAAG
Mouse GLS	CCATCAAGCCTCACATCTCTAC	GTGCAGTTTCGCTGTCTTTAC
Human GDH1	CATGGCTGACTTCCTCACTATC	GGGCTGACTTGGATTGACTT
Mouse GDH1	ATCGGGTGCATCTGAGAAAG	CAGGTCCAATCCCAGGTTATAC
Human GDH2	GGAATGACACCAGGGTTTAGAG	TCAGACTCACCAACAGCAATAC
Human AST	CAACTGGGATTGACCCAACT	GGAACAGAAACCGGTGCTT
Mouse AST	GCGCCTCCATCAGTCTTTG	ATTCATCTGTGCGGTACGCTC
Human GPAT	GTCAGGAGAGTGCTGGTATTG	CCCATTCCCTTGTGTGATTTG
Mouse GPAT	AGTCGCTTCACCACCAATAA	CAGTCCTTCCACTGACAGATAC
Human CPSII	CTGAAGGGATGGAAGGAGATTG	TACGTGACACAGTTGCCATAG
Mouse CPSII	GCTGGCAGACAAGGTCTATTT	ACCATCTGGGCGTTCATTAC
Human β-actin	CGCCGCCAGCTCACCATG	CACGATGGAGGGGAAGACGG
Mouse GAPDH	CATGTTCCAGTATGACTCCACTC	GGCCTCACCCCATTTGATGT

Supplementary Table 1: Glutamine uptake in mouse and human cell lines

Cell lines	Type	K-Ras [a]	TP53 [b]	$\Delta\ ^{120-0}\%U_n$ [c]	P value [d]
HPDE	Control Human Pancreatic Ductal Epithelial cells	Wt	Wt	11,6 ± 2.6	
MDA-MB-231	Human Breast Adenocarcinoma	Mut (p.G13D)	Mut (p.R280K)	2.3 ± 1.52	n.s.
MCF7	Human Breast Adenocarcinoma	Wt	Wt	15.4 + 0.12	n.s.
BT494	Human Breast Adenocarcinoma	Wt	Mut (rear.)	7.0 + 0.08	n.s.
DU4475	Human Breast Adenocarcinoma	Wt	Wt	77.3 + 0.43	***
NCI-H441	Human NSCLS	Mut (p.G12V)	Mut (p.R158L)	23.7 ± 8.65	n.s.
BxPC3	Human PDAC	Wt	Mut (p.Y220C)	12 ± 3,61	n.s.
PT45	Human PDAC	Mut (p.G12C)	Mut (p.R280K)	35.6 ± 4.41	***
MiaPaCa2	Human PDAC	Mut (p.G12C)	Mut (p.R248W)	20.8 ± 4.56	***
Panc1	Human PDAC	Mut (p.G12D)	Mut (p.R273H)	25.7 ± 3.75	***
T3M4	Human PDAC	Mut (p.Q61H)	Mut (p.Y220C)	60.0 ± 5.73	***
Hs766T	Human PDAC	Mut (p.Q61H)	Mut (rearr.)	56.0 ± 5.54	***
CFPAC1	Human PDAC	Mut (p.G12V)	Mut (p.C242R)	62.0 ± 5.83	***
Colo.357-L3.6pl	Human PDAC	Mut (p.G12D)	Wt	57.3 ± 5.60	***
K8484	Mouse PDAC	Mut (p.G12D)	Mut (p.R172H)	72.0 ± 6.28	***
DT4313	Mouse PDAC	Mut (p.G12D)	Wt	32.0 ± 4.23	**
DT6606	Mouse PDAC	Mut (p.G12D)	Wt	52.0 ± 5.35	***

[a-b] Molecular genotypes of analyzed human and murine cell lines. Mutational status was obtained from ATCC (www.atcc.org) and from Cancer Cell Line Encyclopedia (CCLE).

[c] $\%U_n$ delta between 120 min and 0 min ± SEM.

[d] *P <.05; ** P <.01; *** P <.001 significance values, compared to those obtained with the HPDE cell line.

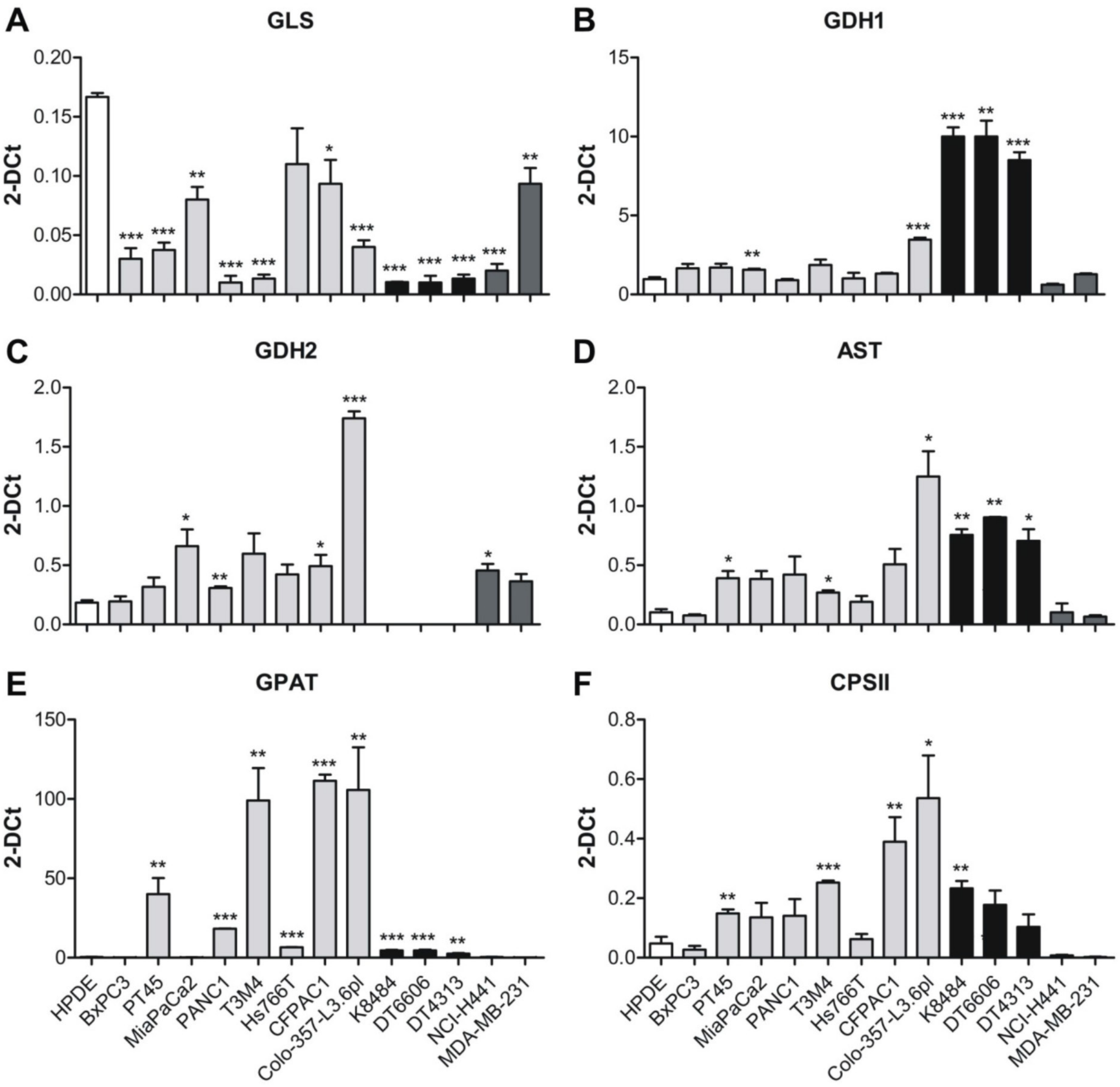

Supplemental Figure 1: PDAC cells rely on glutamine to support nucleotide biosynthesis. **(A-F)** mRNA expression analysis of: GLS (A), GDH1 (B), GDH2 (C), AST (F), GPAT (E) and CPSII (F) in normal pancreatic cells HPDE (white bar), human pancreatic tumor cells BxPC3, PT45, MiaPaCa2, PANC1, T3M4, Hs766T, CFPAC1, Colo-357-L3.6pl (light grey bars), murine pancreatic tumor cells K8484, DT6606, DT4313 (black bars) and non-PDAC cells NCI-H441 and MDA-MB-231 (dark grey bars). mRNA expression levels are represented as 2-DCt and results are expressed as mean±SEM of triplicates of two independent experiments. * P <.05; ** P <.01; *** P <.001 values different from those obtained with the HPDE cell line.

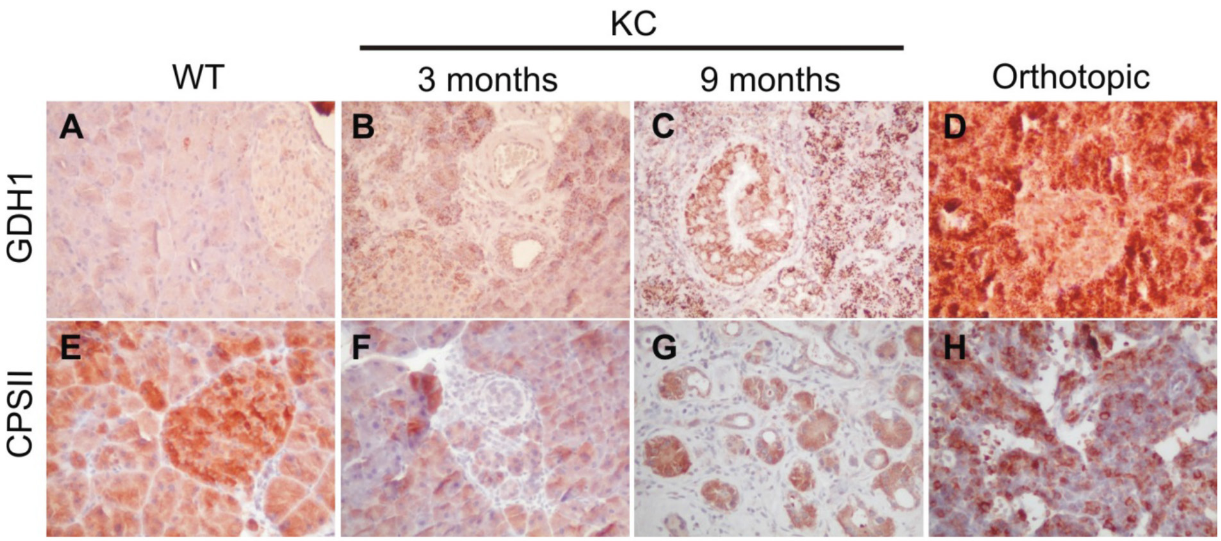

Supplemental Figure 2: GDH1 and CPSII expression correlate with tumor progression. **(A-H)** IHC staining of pancreas sections of control WT mice (A-E), 3 months old (B-F) and 6 months old (C-G) KC mice and of C57BL/6 mice injected orthotopically with K8484 cells. (D-H) Tissues were stained with anti GDH1 (A-D) or anti CPSII (E-H) antibody and examined in a double-blind fashion and digital images of representative areas were taken.

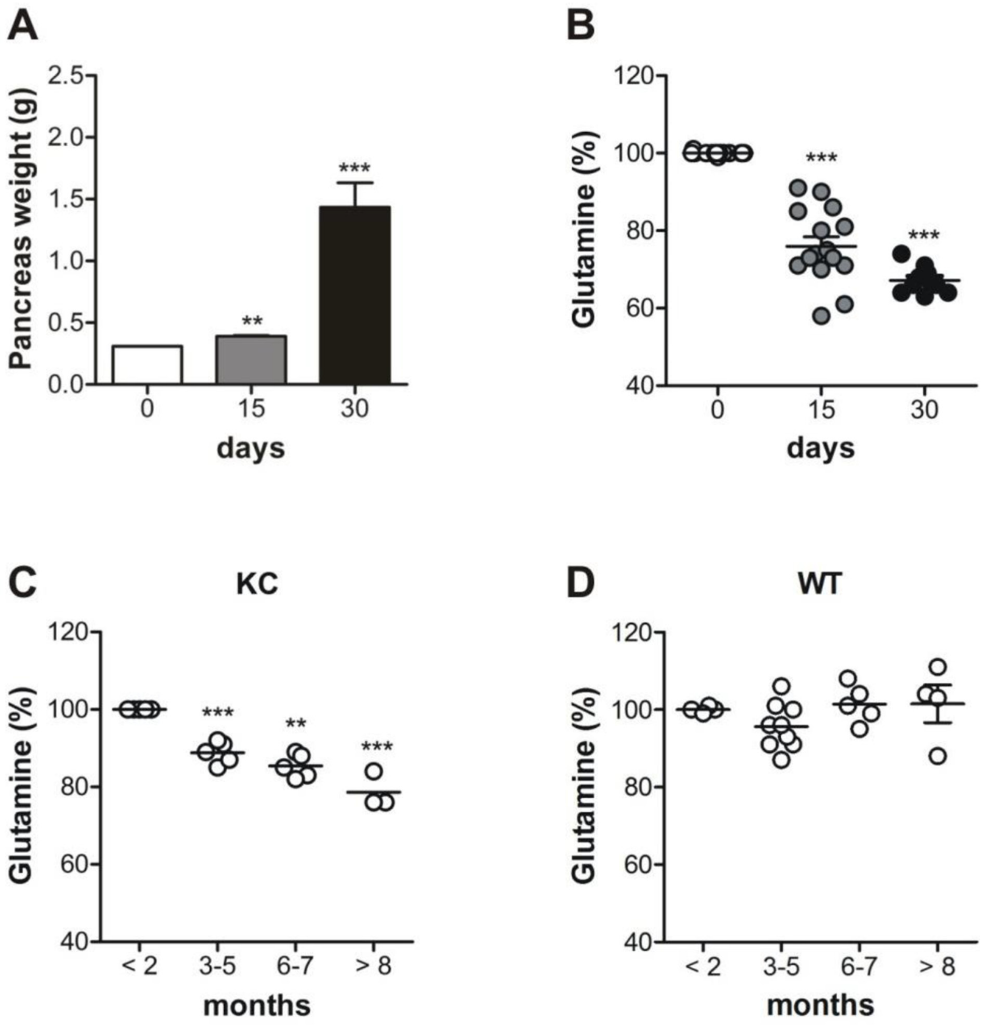

Supplemental Figure 3: *In vivo* glutamine measurement with MS. (A) C57BL/6 mice were injected orthotopically into the pancreas with K8484 cells. Pancreases were collected and weighed at 0, 15 and 30 days after cell injection. **(B)** Graph represents percentage of blood glutamine compared to time 0 in orthotopically injected C57BL/6 mice evaluated using a MS instrument at the indicated time points. Results are represented for each mouse (10-15 mice/time point) and mean±SEM is indicated. **(C-D)** Percentage of blood glutamine in KC mice (C) and WT mice (D) (3-10 mice/group) at indicated time points compared to control group (mice <2 months old) is plotted for each mouse. Mean±SEM is indicated. * $P < .05$; ** $P < .01$; *** $P < .001$ values showing a significant difference of days 15 and 30 compared to day 0 for the orthotopic model, and KC and WT mice older than 2 months compared to mice less than 2 months old.
